# Maternal Prenatal Mental Health and Placental 11β-HSD2 Gene Expression: Initial Findings from the Mercy Pregnancy and Emotional Wellbeing Study

**DOI:** 10.3390/ijms161126034

**Published:** 2015-11-17

**Authors:** Sunaina Seth, Andrew James Lewis, Richard Saffery, Martha Lappas, Megan Galbally

**Affiliations:** 1 School of Psychology, Deakin University, Melbourne 3125, Australia; sunaina@deakin.edu.au; 2 Centre for Social and Early Emotional Development, Deakin University, Melbourne 3125, Australia; 3 Cancer and Disease Epigenetics, Murdoch Childrens Research Institute and Department of Paediatrics, University of Melbourne, Parkville 3052, Australia; richard.saffery@mcri.edu.au; 4 Mercy Perinatal Research Centre, Mercy Hospital for Women, Melbourne 3084, Australia; 5 Obstetrics, Nutrition and Endocrinology Group, Department of Obstetrics and Gynaecology, University of Melbourne, Parkville 3052, Australia; mlappas@unimelb.edu.au; 6 Department of Perinatal Mental Health, Mercy Hospital for Women, Melbourne 3084, Australia; MGalbally@mercy.com.au

**Keywords:** 11-beta hydroxysteroid dehydrogenase type 2, placenta 11β-HSD2, *HSD11B2*, prenatal depression, prenatal anxiety, cortisol, fetal programming

## Abstract

High intrauterine cortisol exposure can inhibit fetal growth and have programming effects for the child’s subsequent stress reactivity. Placental 11beta-hydroxysteroid dehydrogenase (11β-HSD2) limits the amount of maternal cortisol transferred to the fetus. However, the relationship between maternal psychopathology and 11β-HSD2 remains poorly defined. This study examined the effect of maternal depressive disorder, antidepressant use and symptoms of depression and anxiety in pregnancy on placental 11β-HSD2 gene (*HSD11B2*) expression. Drawing on data from the Mercy Pregnancy and Emotional Wellbeing Study, placental *HSD11B2* expression was compared among 33 pregnant women, who were selected based on membership of three groups; depressed (untreated), taking antidepressants and controls. Furthermore, associations between placental *HSD11B2* and scores on the State-Trait Anxiety Inventory (STAI) and Edinburgh Postnatal Depression Scale (EPDS) during 12–18 and 28–34 weeks gestation were examined. Findings revealed negative correlations between *HSD11B2* and both the EPDS and STAI (*r* = −0.11 to −0.28), with associations being particularly prominent during late gestation. Depressed and antidepressant exposed groups also displayed markedly lower placental *HSD11B2* expression levels than controls. These findings suggest that maternal depression and anxiety may impact on fetal programming by down-regulating *HSD11B2*, and antidepressant treatment alone is unlikely to protect against this effect.

## 1. Introduction

Maternal depression and anxiety during pregnancy has been associated with adverse neurodevelopmental outcomes for the offspring, such as attention deficit hyperactivity disorder [1] schizophrenia [2], poor cognitive or emotional developmental [3] and autism [4], although the mechanisms involved remain elusive. Some recent studies [5,6,7,8] examining the role of maternal-fetal cortisol regulation over pregnancy have found that depression and anxiety during pregnancy increases maternal cortisol concentrations, with the intrauterine environment reflecting this elevation [9]. The fetus is typically exposed to 10%–20% of maternal cortisol [10] and high levels *in utero* may hinder neonatal development [11,12]. For example, Bergman *et al.* (2010) [11] found that fetal cortisol exposure (indexed by amniotic fluid levels) negatively predicted cognitive ability in 17 month old infants. However, research investigating the relationship between cortisol and maternal distress is inconsistent [13,14,15] and therefore the suggestion that maternal depression or anxiety enhances the possibility of negative infant outcomes by increasing maternal cortisol levels has not been widely accepted.

11beta-Hydroxysteroid Dehydrogenase type 2 (11β-HSD2), an enzyme highly expressed in aldosterone-selective tissues (distal nephron, colon) and the placenta [16,17], usually limits the amount of maternal cortisol transferred to the fetus by converting cortisol into an inactive metabolite, cortisone [16]. Expression levels of 11β-HSD2 (encoded by the gene, *HSD11B2*) rise in parallel with increasing cortisol levels across pregnancy, protecting the fetus from excess cortisol exposure *in utero* [18,19]. Thus, another theory suggests that 11β-HSD2 gene expression is downregulated by maternal depression or anxiety, resulting in more cortisol being transferred from the mother to the fetus, subsequently contributing to negative infant outcomes.

In support of *HSD11B2* expression mediating the relationship between maternal distress and infant outcomes, a recent study [20] selectively bred Wistar rats for high anxiety-related behaviour or low anxiety-related behaviour and exposed both groups to identical stressors. Those bred for high anxiety had significantly lower placental *HSD11B2* expression levels than the low anxiety group. Additional animal studies on rats also indicate that exposure to chronic stress downregulates *HSD11B2* [21,22,23,24] but report that acute maternal stress upregulates its expression [22,25]. Furthermore, these studies [20,21,23] suggest that the association between maternal anxiety or stress and placental *HSD11B2* is specific to maternal distress during late pregnancy.

Although animal research suggests that *HSD11B2* is affected by maternal stress or anxiety in late gestation and is differentially influenced by chronic *versus* acute symptoms, the applicability of these findings to humans is currently unknown and should be considered in the context of altering levels of maternal distress across pregnancy. For example, there is significant variability in anxiety and depressive symptoms during the perinatal period in humans; some studies report maternal symptoms to be relatively stable during pregnancy [26,27], others identify increasing symptom rates across pregnancy [28] or a u-shaped pattern, where symptoms peak during the first and third trimesters [29,30]. To our knowledge, no study has yet investigated the effects of changing depressive and anxiety symptoms across pregnancy on placental *HSD11B2* expression. However, since animal research [21,23] suggests that placental *HSD11B2* may be influenced by gestation and the chronicity of maternal distress, it is possible that the episodic nature of prenatal depression and anxiety also has an influence on the expression of placental *HSD11B2*.

In human research to date, three studies have investigated the influence of maternal distress on placental *HSD11B2* expression levels, with contrasting findings [31,32,33]. Specifically, O’Donnell *et al.* (2012) [32] and Ponder *et al.* (2011) [31] investigated the effect of both anxiety and depression while Reynolds *et al.* (2015) [33] examined depression alone. In terms of anxiety, O’Donnell *et al*. (2012) [32] found that maternal anxiety in late pregnancy significantly downregulated *HSD11B2* (by approximately 30%) [32]. In contrast, Ponder *et al.* (2011) [31] compared 11β-HSD2 gene expression in women grouped into three categories; those exposed to selective serotonin reuptake inhibitors (SSRI), women with untreated depressive or anxiety symptoms, and a control group. They reported greater expression in the SSRI and depressed/anxious groups in comparison to controls, but this difference did not reach significance. When considering depression alone, O’Donnell *et al.* (2012) [32] found that maternal depression displayed a negative but non-significant association with *HSD11B2* expression while Reynolds *et al.* (2015) [33] reported a positive non-significant association between *HSD11B2* expression and depressive symptoms across all trimesters of pregnancy.

One possible reason for the variations in findings can be attributed to methodological differences. O’Donnell and Reynolds [32,33] used self-report measures to identify maternal depression and anxiety whereas Ponder *et al.* (2011) [31] used retrospective medical records, where a diagnosis of depression or anxiety was elicited by a medical provider or endorsed by the patient at any time during pregnancy. In addition, timing of assessment and placental collection varied across studies. Ponder *et al.* (2011) [31] retrieved antenatal health records early in pregnancy, while Reynolds *et al.* (2015) [33] measured depression across repeated intervals from 12–14 weeks gestation to term. In contrast, O’Donnell *et al.* (2012) [32] administered self-report questionnaires one day prior to the participant’s elective caesarean, when participant responses might have been influenced by their pending surgery and birth. Furthermore, the time taken to process placental tissue differed between studies; O’Donnell *et al.* (2012) [32], Reynolds *et al.* (2015) [33] and Ponder *et al.* (2011) [31] processed samples within 1 h, 90 min and 3 h after delivery, respectively. Collection of the placenta and processing time can effect RNA integrity, with Jobarteh *et al.* (2014) [34] recommending that samples be processed within 90 min of delivery for gene expression studies. Moreover, potential important confounding variables were not consistently controlled for across studies. For instance, smoking and obesity increase norepinephrine in the body [34,35,36], which suppresses *HSD11B2* expression levels [34,35,37]. Likewise, preeclampsia has also been associated with lower levels of *HSD11B2* [38]. O’Donnell *et al.* (2012) [32] did not control for obesity and none of the studies controlled for preeclampsia, with Reynolds *et al.* (2015) [33] specifically including participants at high risk of this condition. Thus, these factors may lower gene expression of 11β-HSD2 in some participants and need to be considered as possible confounders.

The current study aimed to investigate the effect of maternal depressive disorders, symptoms of maternal anxiety and depression across pregnancy on *HSD11B2* expression levels. In doing so, the study also intended to determine whether maternal anxiety or depression symptoms have different effects on *HSD11B2* expression. Since some animal studies have found downregulation of *HSD11B2* specifically during late gestation [21,23], another aim of our research was to investigate whether depressive symptoms and anxiety measured in the first compared to the third trimester of pregnancy have a differential impact on placental *HSD11B2* expression. Furthermore, the effect of changing anxiety and depressive symptoms across pregnancy on placental *HSD11B2* expression was examined and given that only one study to date (Ponder *et al.* 2011 [31]) has investigated the association between antidepressants and *HSD11B2* [39], the impact of antidepressant exposure on placental *HSD11B2* was considered. Lastly, the relationship between *HSD11B2* expression and participant characteristics such as Body Mass Index (BMI), smoking and alcohol consumption has been investigated in this study.

Based on the literature, we hypothesize that: (1) anxiety and depressive symptoms will be negatively associated with *HSD11B2* expression; (2) the effect of anxiety and depressive symptoms on *HSD11B2* will be greater during the third trimester than the first; (3) changes in depression and anxiety symptoms across pregnancy will be inversely related to *HSD11B2* expression levels; (4) in accordance with Ponder *et al.* (2011)’s study [31], anxiety will have a significantly greater effect on *HSD11B2* than depression; (5) smoking, alcohol consumption and BMI will be negatively associated with *HSD11B2* expression levels; and (6) *HSD11B2* expression will be lower in women with a major depressive episode and those exposed to antidepressants than controls [39].

## 2. Results

### 2.1. Preliminary Analyses

In terms of changes in anxiety or depressive symptoms across pregnancy, a paired-samples *t*-test revealed that participant STAI-T (trait anxiety) scores did not significantly or notably differ between Trimester 1 and 3 (*t* (32) = 1.14, *p* = 0.17), remaining relatively stable across gestation. However, a marked positive (although non-significant) improvement in EPDS scores (*t* (32) = 1.85, *p* = 0.07) and a significant improvement in STAI-S (state anxiety) scores between trimesters was found (*t* (32) = 2.41, *p* = 0. 02).

#### Correspondence between the Structured Clinical Interview for DSM-IV Disorders (SCID) and the Edinburgh Postnatal Depression Scale (EPDS)

Out of the nine participants that received a diagnosis of current depression based on the SCID (including one participant that was taking antidepressants), six had an EPDS score of 13 or greater (the validated cut-off score to detect probable cases of major depression [40,41]) in the first trimester and three had a score of 13 or greater in the third trimester. The SCID was administered in Trimester 1 and therefore the greater consistency observed between SCID diagnoses and Trimester 1 EPDS scores (in comparison to Trimester 3 scores) was expected.

### 2.2. Confirmatory Analyses

The first four hypotheses were tested using regression analyses including all participants and the last hypothesis was tested by comparing *HSD11B2* expression levels between participant groups; depressed (untreated), taking antidepressants and controls (see Experimental Section for details).

All statistical analyses remained robust to the exclusion of an outlier in the Trimester 3 EPDS scores and two outliers in the Trimester 3 STAI-S scores.

#### 2.2.1. Hypotheses 1 and 2: The Effects of Self-Reported Maternal Anxiety and Depressive Symptoms on 11β-HSD2 Gene Expression

As presented in Table 1, Pearson correlations showed negative relationships between *HSD11B2* expression and measures of anxiety (STAI-T, STAI-S) in the first and third trimesters of pregnancy. However, although in the predicted direction, these associations did not reach significant alpha levels given the small sample size. These bivariate correlations further revealed a greater effect in the third trimester of pregnancy, where state and trait anxiety in combination accounted for 14% of variance in *HSD11B2* expression levels (*R*^2^ = 0.08 and 0.06 respectively), in comparison to 7% (*R^2^* = 0.01 and 0.06 respectively) in Trimester 1 (see Table 1).

**Table 1:** Pearson Correlations between 11β-HSD2 Gene Expression, Maternal Mood and Participant Characteristics during Trimester 1 and 3.

Participant Variables	Relative 11β-HSD2/L9 Expression (*n* = 33)		
	T1	T3	Differences between T1 and T3
STAI (Trait Anxiety)	−0.24 (*p* = 0.22)	−0.25 (*p* = 0.12)	0.04 (*p* = 0.82)
STAI (State Anxiety)	−0.10 (*p* = 0.64)	−0.28 (*p* = 0.14)	−0.16 (*p* = 0.38)
EPDS	−0.11(*p* = 0.53)	−0.27 (*p* = 0.15)	−0.22 (*p* = 0.21)
Smoking (nicotine consumption)	−0.09 (*p* = 0.60)	−0.07 (*p* = 0.71)	-
Alcohol consumption	−0.17 (*p* = 0.34)	−0.20 (*p* = 0.26)	-
BMI	0.08 (*p* = 0.67)	-	-

STAI = State-Trait Anxiety Inventory; EPDS = Edinburgh Postnatal Depression Scale; T1 = Trimester 1; T3 = Trimester 3; BMI = Body Mass Index.

Pearson correlations also revealed a negative relationship between depressive symptoms (as measured by the EPDS) and *HSD11B2*. Similar to anxiety, the effect was greater in the third trimester than the first, with 7% of the variation in *HSD11B2* being explained by Trimester 3 EPDS scores (in comparison to 1.25% during the first trimester).

#### 2.2.2. Hypothesis 3: Relationship between *HSD11B2* and Changes in Depressive and Anxiety Symptoms across Pregnancy

**Figure 1:**
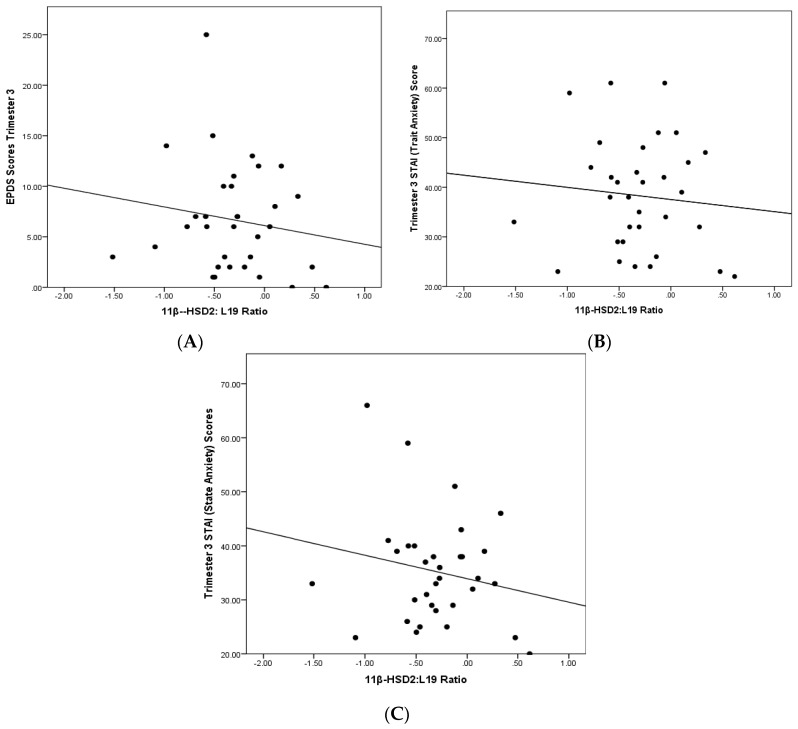
Placental *HSD11B2* expression and Trimester 3 (**A**) EPDS scores (*M* = 6.22, *SD *= 4.50); (**B**) STAI-T scores (*M* = 36.48, *SD* = 10.38); and (**C**) STAI-S scores (*M* = 34.35, *SD* = 10.30).

Correlations between *HSD11B2* expression and changes in scores on the EPDS and STAI-S (state anxiety) between the first and third trimester suggested negative relationships (see Table 1), while the correlation between *HSD11B2* and changes in STAI-T (trait anxiety) remained positive (see Figure 1).

#### 2.2.3. Hypothesis 4: Comparison between Anxiety and Depression; Strength of their Association with *HSD11B2*

Fischer’s R-Z transformations were used to test the strength of association between maternal trait anxiety and placental *HSD11B2*, relative to other measures of maternal mood. In relation to the strength of their association with *HSD11B2*, results indicated that maternal trait and state anxiety in both Trimester 1 and 3 did not significantly differ from each other. Likewise, the correlations between maternal depression and *HSD11B2* expression were are not significantly different from associations between *HSD11B2* expression and state or trait anxiety in either trimester.

#### 2.2.4. Hypothesis 5: The Influence of Alcohol Consumption, Smoking and Body Mass Index on *HSD11B2*

Pearson correlations revealed a negative effect of alcohol consumption and smoking on *HSD11B2* expression levels, while a positive correlation was found between BMI and *HSD11B2* (see Table 1). An Analysis of Covariance (ANCOVA) indicated that BMI in Trimester 1 of pregnancy contributed to 0.2% of the relationship between *HSD11B2* and group status, while alcohol in Trimester 3 accounted for 5% of this relationship. Meanwhile, smoking in Trimester 3 of pregnancy showed a negligible (η_p_^2^ = 0.002, *p* = 0.53) effect on *HSD11B2*.

#### 2.2.5. Hypothesis 6: Comparison of *HSD11B2* Expression Levels between Groups

**Figure 2:**
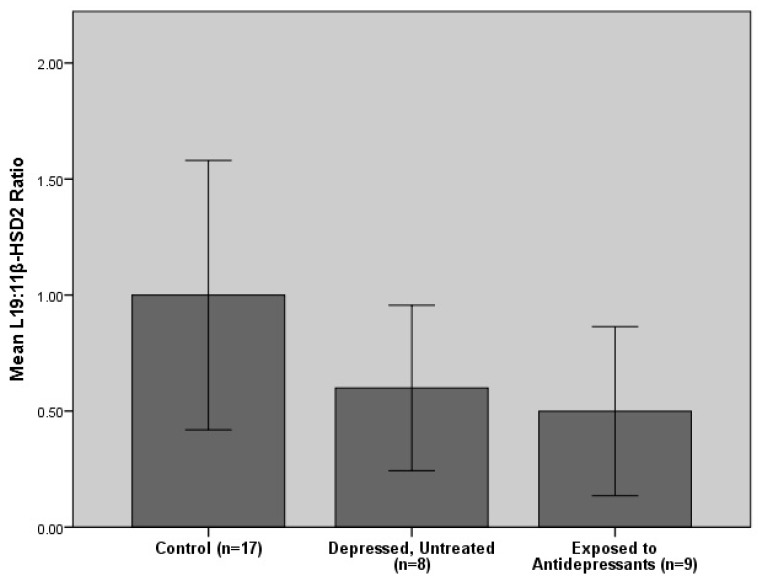
Comparisons of placental *HSD11B2* expression levels between the control, depressed (untreated) and antidepressant exposed groups. Histogram shows mean values +/− standard error of the mean.

An ANCOVA controlling for BMI, smoking and alcohol consumption revealed that group status (antidepressant exposure, untreated depression and controls) accounted for 5.9% of *HSD11B2* expression (*F* (2, 32) = 0.84, *p* = 0.44, *n* = 33) instead of 7.1% (the computed effect size without consideration of covariates). The ANCOVA also revealed that participants with untreated depression and antidepressant exposure had markedly lower *HSD11B2* concentrations than controls (refer to Figure 2). Excluding the participant with limited antidepressant exposure in this analysis produced almost identical results (*F* (2, 31) = 0.85, *p* = 0.44, *n* = 32), indicating that the findings are robust to this sensitivity analysis. 

## 3. Discussion

This pilot study was broadly in line with predictions and produced a number of important and novel findings. Although none of our results were statistically significant due to a small sample size, the effect sizes and direction of associations are of interest and indicate meaningful findings. First, we found negative associations between *HSD11B2* expression and self-reported maternal anxiety and depression. Likewise, changes in self-reported maternal state anxiety and depressive symptoms from Trimester 1 to 3 were negatively related to *HSD11B2*, where improvements in maternal mental health (lower EPDS and STAI scores) led to higher placental *HSD11B2* expression levels. As an exception, the relationship between trait anxiety and *HSD11B2* did not differ considerably between trimesters; however this is consistent with the nature of trait anxiety, which is relatively stable across time. 11β-HSD2 limits the amount of cortisol that is transferred from the mother to the fetus [16] and therefore, these results suggest that maternal anxiety and depressive symptoms may increase fetal cortisol exposure.

Furthermore, maternal anxiety and depression measured in late gestation displayed stronger associations with placental *HSD11B2* expression levels than maternal depression and anxiety in early pregnancy (12–18 weeks). This is consistent with previous animal research [21,23] and the human study of O’Donnell *et al.* (2012) [32], where maternal anxiety was measured one day before term and exhibited a significant positive association with the downregulation of *HSD11B2*. These findings are also noteworthy because maternal cortisol levels are usually at their peak during the third trimester of pregnancy [42]. Although it might be suggested that the association between *HSD11B2* and maternal distress is stronger in the third trimester due to worsening of anxiety and depressive symptoms across pregnancy, our results indicate a significant improvement in state anxiety and a notable improvement (although non-significant) in depressive symptoms, from Trimester 1 to 3 [43]. Thus, based on our findings and O’Donnell’s research [32], it is suggested that elevated maternal cortisol accompanied with lowered *HSD11B2* expression levels during late gestation may be sufficient to adversely affect fetal development, reflecting a possible mechanism between maternal distress and fetal programming.

The results further indicate that women with untreated major depression and those using antidepressants have markedly lower placental *HSD11B2* expression levels than controls. However, a notable difference in placental *HSD11B2* expression was not evident between women taking antidepressant medication and those with untreated depression, which may suggest that antidepressant treatment alone is not effective in reversing the effects of maternal depression and anxiety on *HSD11B2* expression. These findings are consistent with our hypotheses and the findings of O’Donnell *et al.* (2012) [32], which found a 30% reduction in placental *HSD11B2* expression in anxious pregnant women and a negative (although non-significant) association between maternal depression and *HSD11B2*. In contrast to O’Donnell *et al.* (2012) [32] however, maternal anxiety did not have a greater effect on *HSD11B2* than depression in our study. Furthermore, our results disagree with Reynolds *et al.* (2015) [33] and Ponder *et al.* (2011) [31], where antidepressant exposure and self-reported maternal depression were found to upregulate placental *HSD11B2* expression levels.

The variation in findings can be attributed to a range of limitations and methodological differences between studies related to outcome measures, time of processing samples and the gestational period at which anxiety and depression were assessed. Furthermore, findings may be influenced by whether chronic or acute depressive or anxiety symptoms were measured. Acute stress in pregnant rats has been found to increase placental 11β-HSD2 enzyme activity [22,24] and betamethasone treatment (which rapidly increases maternal cortisol levels) within 72 h of birth has also been reported to increase 11β-HSD2 enzymatic activity in postpartum women [44]. In contrast, animal research has indicated that chronic stress lowers *HSD11B2* expression in the placenta [20,21,22,23,25]. Thus, it is possible that acute depression or anxiety upregulates *HSD11B2* while chronic depression or anxiety has the opposite effect. This important point of difference requires further investigation.

It can be argued that the negative association between maternal distress and *HSD11B2* expression found in this study supports the premise that acute stress upregulates while chronic stress downregulates *HSD211B2*, since major depressive disorders were measured (using the SCID) rather than situational distress and most participants that screened positive for antenatal depression have a history of depression prior to pregnancy. Furthermore, studies (Ponder *et al.* 2011 [31] and Reynolds *et al.* 2015 [33]) reporting a positive relationship between *HSD11B2* and maternal mood used medical records and the CES-D as a basis for diagnosis, arguably measuring less severe or chronic symptomology than the SCID. Although our findings also report a negative relationship between *HSD11B2* and self-reported maternal anxiety and depression (using the EPDS and STAI), a study [45] comparing the EPDS and CES-D found that the EPDS is a better predictor of major depression and attuned to greater symptom severity than the CES-D. In addition, majority (67%) of participants diagnosed with a major depressive episode on the SCID obtained an EPDS score of 13 or greater in our study and Reynolds *et al.* (2015) [33] only identified 12.5%–16.3% of participants as exhibiting high CES-D scores. Thus, it is possible that human research also lends support to the idea that downregulation of *HSD11B2* expression is related to chronic depression or anxiety and upregulation is associated with acute distress.

Theoretically, it is suggested that *HSD11B2* expression levels increase in women with acute stress to levels greater than normal (as seen in the findings of Reynolds and Ponder), since the placenta aims to protect the fetus from excess glucocorticoids produced by mothers during this period [21]. In contrast, chronic stress may be characterized by hypocortisolemia (insufficient production of cortisol) [46] or epigenetic changes in the placenta, leading to the downregulation of *HSD11B2*. Although the exact mechanism between maternal distress and downregulation of *HSD11B2* is unknown [21], elevations in proinflammatory cytokines [47,48], stimulation of the sympathoadrenal system [49] and DNA methylation [21] have been implicated. However to date, no human study has differentiated between the effects of acute, chronic, mild or severe depression or anxiety on *HSD11B2* expression, and greater research investigating the effect of distress severity and chronicity on *HSD11B2* is needed to test the suggested hypotheses.

### 3.1. The Influence of Smoking, BMI and Alcohol Consumption

The current study also explored the effect of smoking, BMI and alcohol consumption on *HSD11B2.* The results indicated a negative relationship between smoking and alcohol consumption, which is consistent with literature on the influence of norepinephrine (in nicotine) on *HSD11B2*. However, there is insufficient research examining the relationship between alcohol and *HSD11B2*, with the current study providing some direction. In contrast, a positive but non-significant association between the first trimester *HSD11B2* expression levels and participant BMI was found. This is inconsistent with the hypothesis that BMI increases norepinephrine which in turn, lowers *HSD11B2* levels. However, these results are not surprising since a direct effect of obesity on *HSD11B2* is unknown and a range of factors may influence the obesity-norepinephrine- *HSD11B2* interaction sequence.

### 3.2. Limitations and Strengths

The study (being a pilot study) has limited its sample size and subsequently, statistical power. In addition, although six out of nine participants with a SCID diagnosis of depression scored 13 or greater on the EPDS in Trimester 1, three participants in the depressed group did not, which suggests some inconsistency in the screening of depressive symptoms in our study. Furthermore, participant BMI was measured between 12–18 weeks gestation (which is not an accurate representation of a woman’s BMI) and similar to all other studies, some suppressants of *HSD11B2* expression such as liquorice and carbenoxolone [50] were not controlled for. However, participants with preeclampsia were excluded and the three groups formed (antidepressant exposed, untreated depressed and controls) were matched on BMI and gestation. Another strength of this study is that associations between *HSD11B2* and changes in maternal distress across pregnancy were examined and the effect of antidepressant exposure on *HSD11B2,* which has only ever been investigated by one other study (Ponder *et al.* 2011 [31]), was considered. Furthermore, depression was measured by both self-report measures and the SCID for DSM-IV, which allows for screening of both depressive symptoms and major depression. In addition, placental samples were collected and processed within a half hour of delivery, ensuring sampling quality. Although numerous procedural advantages can be identified in the current research, due to the limited sample size, this study is primarily exploratory.

### 3.3. Lessons Learned in Current Study and Future Directions

Given the limitations of this study, it is suggested that future research;

1. Include an adequate sample size. A *post hoc* power analysis revealed that *n* = 193 participants are required to conduct the analyses in this study and obtain statistical power of 0.80, with a medium effect size of *r* = 0.20 and alpha significance level of 0.05. A further 58 participants (total *n* = 251) are needed (30% of 193) if covariates (smoking, alcohol consumption and BMI) are included in the analysis [51].
2. Control for major suppressants of *HSD11B2* such as liquorice and carbenoxolone.
3. Participant BMI values in this study may be inaccurate as they were based on measurements during pregnancy. In future studies, the pre-pregnancy BMI of women should be obtained.
4. The current study used a structured interview to assess depression, but not anxiety. To enhance our study design, a semi-structured or structured interview could have been used to measure both constructs.
5. The current study had one participant that discontinued antidepressant medication during the first 11 weeks of pregnancy. To cater for this, we conducted a sensitivity analysis excluding this participant from the statistical analyses computed. However, ideally, all participants in the antidepressant group should have consistent exposure to antidepressants across pregnancy to avoid individual variability that can potentially bias results.
6. To optimally assess depression, anxiety and stress in pregnancy, the EPDS and STAI should be administered during each trimester. Furthermore, a diagnostic assessment of DSM-V depression, anxiety and stress related disorders should be conducted during the beginning and end of pregnancy. Although there is currently no diagnostic measure designed specifically for pregnancy, assessment tools such as the SCID have been widely used in psychiatric research studies [52]. The diagnostic measure at the start of pregnancy should assess both current and lifetime mental health disorders while the diagnostic measure at the end of pregnancy should assess onset and course of disorders occurring during pregnancy as well as the continuity/discontinuity of pre-existing disorders. It would also be important in this protocol to rule out other classes of mental disorder.

Furthermore, to expand on the preliminary work conducted in this study, the finding that antidepressant treatment alone does not normalize *HSD11B2* expression levels needs to be explored further, as this can contribute to understanding the impact of maternal depression and anxiety on placental 11β-HSD2 enzymatic activity.

## 4. Experimental Section

### 4.1. Participants

This research is part of a larger cohort study known as the Mercy Pregnancy and Emotional Wellbeing Study (MPEWS). This study reports on a selection of 33 participants who were recruited from the Mercy Hospital for Women (Melbourne, Australia) during their first trimester (between 12 and 18 weeks’ gestation). Women with gestational diabetes mellitus (GDM) or preeclampsia were excluded. As part of the larger MPEWS study, written informed consent, weight, height and contact details were obtained from all participants at the time of recruitment.

At the time of initial contact in the first trimester, a questionnaire was administered which included the Edinburgh Postnatal Depression Scale (EPDS), the State-Trait Anxiety Inventory (STAI) and questions about the participants smoking status, exposure to antidepressants and alcohol consumption. Furthermore, participants were assessed for a major mood disorder using the Structured Clinical Interview for DSM-4 Disorders (SCID) during their first trimester and those who screened positive for mania, bipolar disorder or psychotic disorders were excluded from the study. A second questionnaire incorporating the same information as the first questionnaire was provided to participants via mail and completed between 28–34 weeks gestation.

Participants were categorized into one of three groups; those exposed to antidepressants, participants with a major depressive disorder (without antidepressant exposure) and a control group (women with neither depression nor exposure to antidepressants). Participants were diagnosed with depression based on the SCID assessment and participant exposure to antidepressants was derived from their first questionnaire responses. Based on this assessment, there were eight participants with current major depression, eight exposed to antidepressants during their pregnancy, and 17 controls. One participant exposed to antidepressants was also diagnosed with current depression and another two participants within the antidepressant exposed group were diagnosed with depression in partial remission. The selection of controls was based on the development of a propensity score match technique so that controls were matched to the two clinical groups on gestational age at the time of delivery and maternal BMI during the first trimester of pregnancy, with participant BMI being calculated by using the following formula: weight (kg)/height^2^ (m^2^). Medications in the antidepressant group included serotonergic antidepressants including selective serotonergic reuptake inhibitors (SSRIs) and serotonin-norepinephrine reuptake inhibitors (SNRIs); five participants used sertraline (50–150 mg daily), one used Duloxetine (120 mg daily), one used Venlafaxine (75 mg daily) and one participant used Fluoxetine (10 mg daily).

### 4.2. Measures of Depression

#### 4.2.1. The Structured Clinical Interview for DSM-IV Disorders

The Structured Clinical Interview for DSM-IV Axis I disorders (SCID-I) is a well-known semi-structured interview designed to detect DSM-IV axis I disorders [53]. It is divided into separate modules corresponding to categories of diagnoses. Interviews were conducted by registered psychologists or postgraduate trainee psychologists.

#### 4.2.2. Edinburgh Postnatal Depression Scale

The Edinburgh Postnatal Depression Scale is a 10-item self-rating scale, developed by Cox *et al.* (1987) [42] to detect perinatal depression. The reliability of the EPDS ranges from a Cronbach’s α of 0.82 to 0.84 [54]. It has demonstrated high concurrent [54] and retest validity [55,56], and studies report the measure as highly valid for detecting depression in a pregnant population [57,58]. A score of 13 or above on this scale is used to identify women at risk of a depressive disorder [41].

### 4.3. Measures of Anxiety

#### State-Trait Anxiety Inventory

The State-Trait Anxiety Inventory is a commonly used measure of trait and state anxiety. It includes 40 items in total, with 20 assessing trait anxiety (STAI-T) and the remaining 20 assessing state anxiety (STAI-S). All items are rated on a four point Likert scale. Internal consistency coefficients for the scale range from 0.89 to 0.91 [59] and considerable evidence attests to the construct and concurrent validity of the scale [59].

### 4.4. 11β-HSD2 Gene (HSD11B2) Expression

Tissues were collected and processed within 30 min of delivery. Using a biopsy punch samples were obtained at six different sites across the placenta for every participant, washed extensively with phosphate-buffered saline, and immediately snap frozen in liquid nitrogen and stored at −80 °C until extracted for RNA. Six different sites were chosen to obtain an accurate representation of the whole placenta. Total RNA was extracted using TRIsure reagent according to manufacturer’s instructions (Bioline; Alexandria, NSW, Australia), as previously described [60]. RNA concentration and purity were measured using a NanoDrop ND1000 spectrophotometer (Thermo Fisher Scientific; Scoresby, Australia). RNA was converted to cDNA using the SuperScript VILO cDNA synthesis kit (Invitrogen, Carlsbad, CA, USA) according to the manufacturer’s instructions. Semi-quantitative Real Time (RT)-PCR was carried out using the SYBR Green method (Bioline; Alexandria, Australia) with primer annealing temperatures set to 60 °C. Placenta 11β-HSD2 gene expression analyses were assayed in duplicate and normalized to the ribosomal protein L19, as detailed in O’Donnell *et al.* (2012) [32], using the following primer sequences: 11β-HSD2 forward: 5’-GCTCATCACCGGGTGTGACT-3′, reverse: 5’-GGGCTGTTCAACTCCAATACG-3′, L19 forward: 5’-CCAACTCCCGTCAGCAGATC-3′, reverse: 5’-CAAGGTGTTTTTCCGGCATC-3′.

### 4.5. Statistical Analysis

Statistical analyses were conducted using SPSS statistical software (version 22.0; SPSS Inc., Chicago, IL, USA). Missing data on one item of the wave 2 STAI-T for one participant was replaced with equivalent item scores from their wave 1 STAI-T scale scores, since trait anxiety was relatively stable across time in our study. Furthermore, the data for relative 11β-HSD2/L19 expression was not normally distributed and all analyses included the bootstrap function (×1000 samples) to cater for this. Graphs were also constructed using log transformed data to address non-normality.

To test the hypothesis that *HSD11B2* shares a negative relationship with maternal distress, correlations between 11β-HSD2 mRNA levels and scores on anxiety and depression self-report measures (EPDS and the STAI) were computed. These relationships are presented graphically (refer to Figure 1). Likewise, to test the association between changes in maternal distress and *HSD11B2,* differences in EPDS and STAI scores between Trimester 1 and 3 were calculated and correlations between these differences and *HSD11B2* expression were computed. Differences in participant EPDS and STAI scores were obtained by subtracting first trimester scores from third trimester scores.

To determine whether anxiety or depression symptoms had a greater effect on *HSD11B2* expression, Fischer’s R-Z transformations were calculated to compare correlation coefficients, using the following formula:

*Z* = 0.5loge [(1 + *r*)/(1 − *r*)] (1)

If the *Z* value was greater or equal to 1.96 or less than or equal to −1.96, the two correlations being compared were considered significantly different at the *p* < 0.05 level of significance.

Pearson correlations between *HSD11B2* and participant characteristics such as; BMI, smoking and alcohol consumption were calculated to investigate these relationships.

In order to examine the hypothesis that placental 11β-HSD2 mRNA levels are lower in women with a major depressive disorder and antidepressant exposed women, we used an Analysis of Covariance (ANCOVA) with participant group (untreated participants with depression, participants using antidepressants and controls) as the independent variable and 11β-HSD2 mRNA levels as the outcome variable and then repeated the analysis controlling for nicotine use (in Trimester 3), alcohol consumption (in Trimester 3), and BMI (in Trimester 1).

Furthermore, seven out of eight participants were exposed to antidepressants for majority of their pregnancy while one participant used antidepressants only during the first 11 weeks of pregnancy. Sensitivity analyses were performed, excluding the participant with limited antidepressant exposure and participants with very high Trimester 3 EPDS and STAI-S scores (outliers). Outliers were identified using boxplots and confirmed using the outlier labeling rule [61].

Lastly, for future research, a power analysis to determine an adequate sample size to test our hypotheses was computed; using the software program G*Power 3.1 [62], an estimated sample size was derived. To cater for covariates, an additional 30% of this estimated sample size was added to form the total number of participants required.

## 5. Conclusions

In conclusion, the current study provides further evidence that placental 11β-HSD2 gene expression is sensitive to both maternal antenatal anxiety and depression, particularly during the third trimester of pregnancy. It is then hypothesized that the influence of maternal anxiety and depression on 11β-HSD2 gene expression may act as a mediator between maternal distress and cortisol exposure *in utero*, consistent with a mechanistic role in mediating the fetal programming effects of maternal depression and anxiety on offspring outcomes. Furthermore, the study suggests that antidepressant treatment and untreated depression have similar effects on *HSD11B2*. However, given the limitations of all studies within this field (including the current study) and gaps within the mechanistic understanding of maternal mood’s effect on *HSD11B2* expression, further research needs to be conducted.
